# The α-Gal mammalian meat allergy as a cause of isolated gastrointestinal symptoms

**DOI:** 10.3389/fgstr.2022.987713

**Published:** 2022-09-23

**Authors:** Nathan E. Richards, Tom A. Makin, Anna R. Smith, Thomas A. E. Platts-Mills, Robert D. Richards, Jeffrey M. Wilson

**Affiliations:** ^1^ Division of Allergy and Clinical Immunology, University of Virginia, Charlottesville, VA, United States; ^2^ Gastroenterology Associates of Central Virginia, Lynchburg, VA, United States

**Keywords:** alpha-gal, alpha-gal syndrome, food allergy, IgE, GI-AGS, IBS, GERD

## Abstract

The α-Gal mammalian meat allergy classically presents with urticaria, with or without gastrointestinal (GI) symptoms or anaphylaxis, but increasingly we are aware of patients with only GI symptoms. Here we describe patients presenting with isolated GI symptoms who had detectable IgE antibodies to α-Gal and reported symptom improvement on a mammal-restricted diet. Forty patients in the practice of a single gastroenterologist, and 35 patients in one allergy clinic were identified, with abdominal pain, diarrhea, and nausea the most common symptoms. Alpha-Gal IgE levels were lower than in a previously described cohort of patients who exhibited classic allergic reactions. This large case series suggests that α-Gal IgE is an important contributor to GI morbidity in areas where lone star tick bites are common. Symptom presentations in GI-AGS can be easily confused with other common GI conditions, and α-Gal IgE levels are often lower than those in patients with classic AGS.

## Introduction

A syndrome of mammalian meat allergy, caused by IgE antibodies specific for the oligosaccharide galactose-α-1,3-galactose (α-Gal), was first described in 2009 (1, 2). The α-Gal syndrome (AGS) often impacts adults who have previously tolerated mammalian meat and characteristically has a delay of several hours between meat ingestion and symptom onset, both features that can contribute to diagnostic confusion and delay in formal diagnosis (3). Tick bites are the dominant mode of α-Gal IgE sensitization, particularly bites from the lone star tick (*Amblyomma americanum)* in North America (4). Accordingly, AGS prevalence is highest in a large area of the eastern and central United States where the lone star tick is established (5). Urticaria and pruritus are classic manifestations of AGS, however many patients develop concurrent gastrointestinal (GI) symptoms and recent reports suggest that isolated GI symptoms may be an underappreciated aspect of the syndrome (6–10). Here we sought to further investigate “GI-variant” AGS (GI-AGS) cases, focusing on clinical and immunologic characteristics of these patients.

## Methods

Retrospective chart review was carried out, using inclusion criteria of: i) isolated GI symptoms in the absence of urticaria, severe pruritus or anaphylaxis, ii) evidence of α-Gal IgE sensitization, and iii) subjective improvement on a mammalian avoidance diet that was adopted after initial testing. Charts of patients seen at an academic allergy clinic and the patients of one gastroenterologist at a private GI clinic in Central Virginia that were seen between September 2019 and June 2021 were reviewed.

Charts were reviewed for demographic data including age and sex, α-Gal IgE levels, symptoms of pain/cramping, nausea, diarrhea, vomiting and timing of symptom onset. Previous diagnoses of gastro-esophageal reflux disease (GERD), irritable bowel syndrome (IBS), celiac disease and “other” were recorded. History of other food allergy, asthma, allergic rhinitis, and history of tick bite were recorded. Frequency of abdominal CT scan, upper endoscopy, and gastroenterology evaluation were recorded. Categorical variables were compared with Chi Squared test and continuous variables were compared with Student’s T test or Mann-Whitney U test, as appropriate. A comparison cohort of 245 patients with “classic” AGS who had experienced urticaria and/or anaphylaxis was previously reported (6).

## Results

Of the 35 cases identified in the allergy practice and 40 cases in the GI practice the median age was 63 years and 64% were women (**Table 1**). The dominant presentation involved abdominal pain (83%), with cramping specifically identified in many cases. The majority also reported diarrhea (53%), with nausea (33%) and vomiting (9%) being less frequent. Timing of symptoms in relation to a meal with mammalian meat was variable and the majority did not recognize mammalian meat as a culprit prior to diagnosis. Many of the patients (55%) reported avoiding dairy in addition to mammalian meat. When compared with patients who presented to the GI clinic, patients seen in the allergy clinic were more likely to report: i) episodes of GI pain/cramping (94% vs 73%), ii) symptoms occurring within 6 hours of eating mammalian meat (51% vs 13%) and iii) history of tick bites (97% vs 53%). Many of these patients had other prior or concomitant GI diagnoses, as listed in **Table 1**.

**Table 1:** Characteristics of patients with diagnosis consistent with GI-variant α-Gal syndrome.

	Characteristics*	Total Patients (n = 75)	Allergy clinic (n = 35)	GI clinic(n = 40)	p
Demographics	Age, median (range)	63 (7-88)	54 (7-74)	67 (24-88)	<0.001
	Sex, female	48 (64%)	19 (54%)	29 (73%)	0.10
Presenting GI symptoms	Pain/cramping	62 (83%)	33 (94%)	29 (73%)	0.01
	Nausea	25 (33%)	17 (49%)	9 (23%)	0.02
	Diarrhea	40 (53%)	20 (57%)	20 (50%)	0.54
	Vomiting	7 (9%)	5 (14%)	2 (5%)	0.17
Timing to symptom onset following ingestion	<2 hours	10 (13%)	7 (20%)	3 (8%)	0.11
	2 to 6 hours	13 (17%)	11 (31%)	2 (5%)	0.003
	>6 to 24 hours	6 (8%)	5 (14%)	1 (3%)	0.06
	chronic/ill-defined	45 (60%)	12 (34%)	33 (83%)	<0.001
Diet	Mammalian meat avoidance	75 (100%)	35 (100%)	40 (100%)	NA
	Dairy avoidance	41 (55%)	22 (63%)	19 (48%)	0.18
Prior/concomitant GI Diagnosis	Gastroesophageal Reflux Disease (GERD)	23 (31%)	11 (31%)	12 (30%)	0.89
	Irritable bowel syndrome (IBS)	10 (13%)	6 (17%)	4 (10%)	0.36
	Celiac Disease	2 (3%)	1 (3%)	1 (3%)	0.92
	Other	17 (23%)	7 (20%)	10 (25%)	0.61
Co-morbid Allergic diseases	Other food allergy	5 (7%)	4 (11%)	1 (3%)	0.12
	Asthma	7 (9%)	4 (11%)	3 (8%)	0.56
	Allergic rhinitis	21 (28%)	13 (37%)	8 (20%)	0.10
Other features of clinical history	History of tick bite	55 (73%)	34 (97%)	21 (53%)	<0.001
	Evaluated by GI doctor	56 (75%)	16 (46%)	40 (100%)	<0.001
GI tests as part of work-up	CT Abdomen	20 (27%)	7 (20%)	13 (33%)	0.22
	Upper endoscopy	27 (36%)	10 (29%)	17 (43%)	0.21
Antibody levels	α-Gal IgE, kU/L, median (IQR)	1.7 (0.4-4.6)	3.3 (1.4-8.5)	0.7 (0.3-2.0)	<0.001

*values in parentheses are reported as % unless otherwise noted.

**Figure 1:**
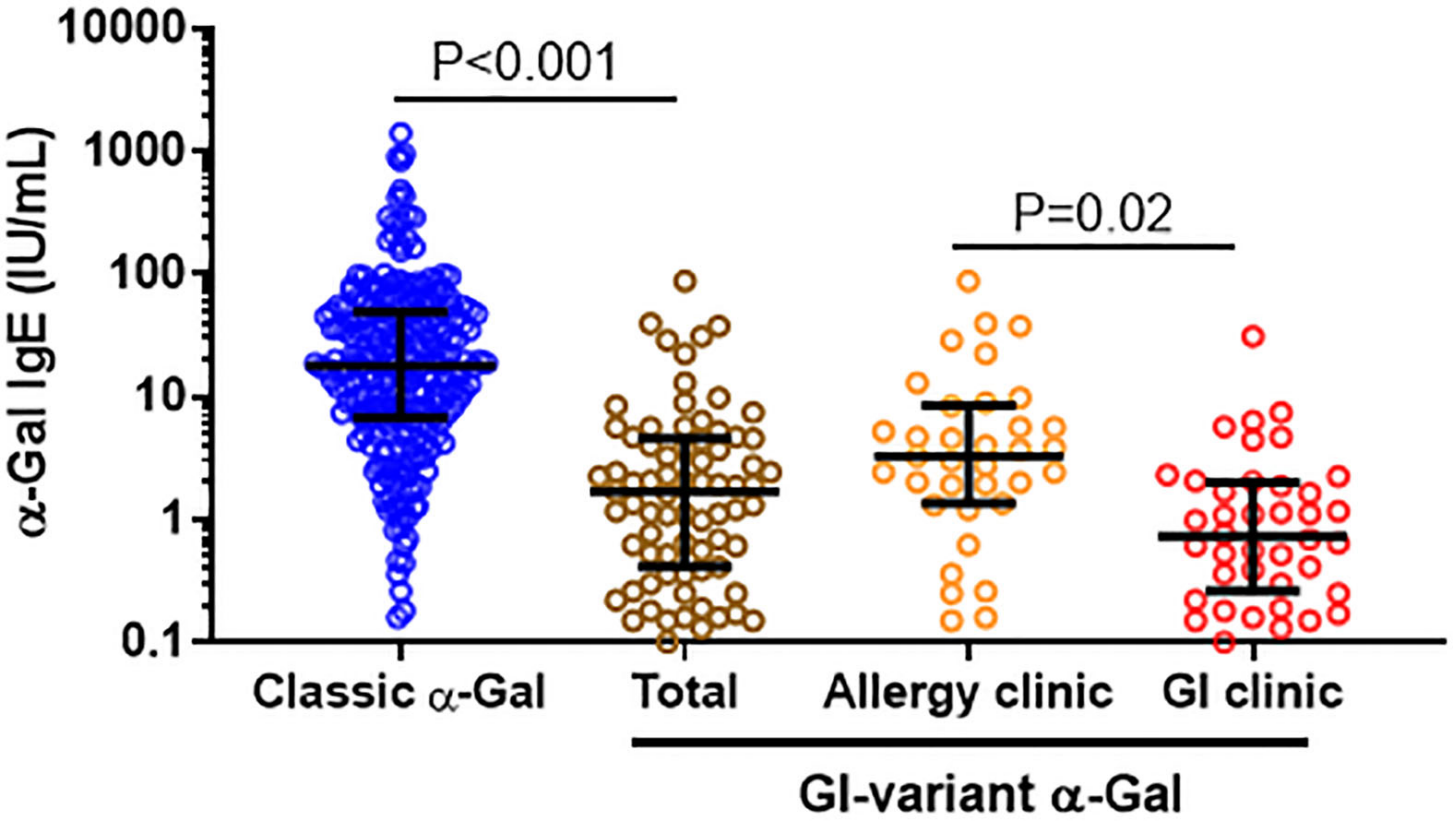
Comparison of α-Gal IgE among GI variant and classic AGS cases. Levels of IgE to α-Gal among patients who met criteria for GI-AGS in an allergy clinic and a gastroenterology practice, as compared to a reference cohort of patients who had classic allergic manifestations of AGS.^6^ Patients considered sensitized to α-Gal with IgE level ≥0.1.

Levels of IgE to α-Gal in these 75 patients were lower than in a previously described cohort of 245 patients with “classic” AGS who had urticaria and/or anaphylaxis (GM 1.7 kU/L [95%CI 0.4-4.6] vs GM 16.8 kU/L [95%CI 13.8-20.5], p<0.001) (**Figure 1**) (6). Compared to the 35 patients presenting to the allergy clinic (GM 3.3 kU/L [95%CI 1.4-8.5]), the 40 patients presenting to the GI clinic had lower levels of IgE to α-Gal (GM 0.7 kU/L [95%CI 0.3-2.0]), P=0.02.

## Discussion

Here we report a case series of 75 patients with symptoms, lab findings and dietary response that are consistent with GI-AGS. Importantly, these patients with unexplained abdominal pain and diarrhea might have otherwise been diagnosed with functional abdominal pain or diarrhea-predominant IBS. Consistent with the prior report by Richards and Richards, GI-AGS can be superimposed on a pre-existing gastrointestinal tract problem, a concomitantly diagnosed GI illness or can be the lone cause for GI symptoms (9). In this study 31% of patients had a pre-existing diagnosis of GERD, 23% other GI diseases, 13% IBS, 3% Celiac. Notably, only 36% underwent upper endoscopy and 27% had a CT scan of the abdomen. We think this reflects changes in the diagnostic paradigm that has occurred in our clinics with earlier testing for α-Gal, as we have become more familiar with GI-AGS as a possible culprit for unexplained GI symptoms. It may also reflect more α-Gal awareness in the greater medical and patient communities leading to earlier diagnosis.

**Figure 2:**
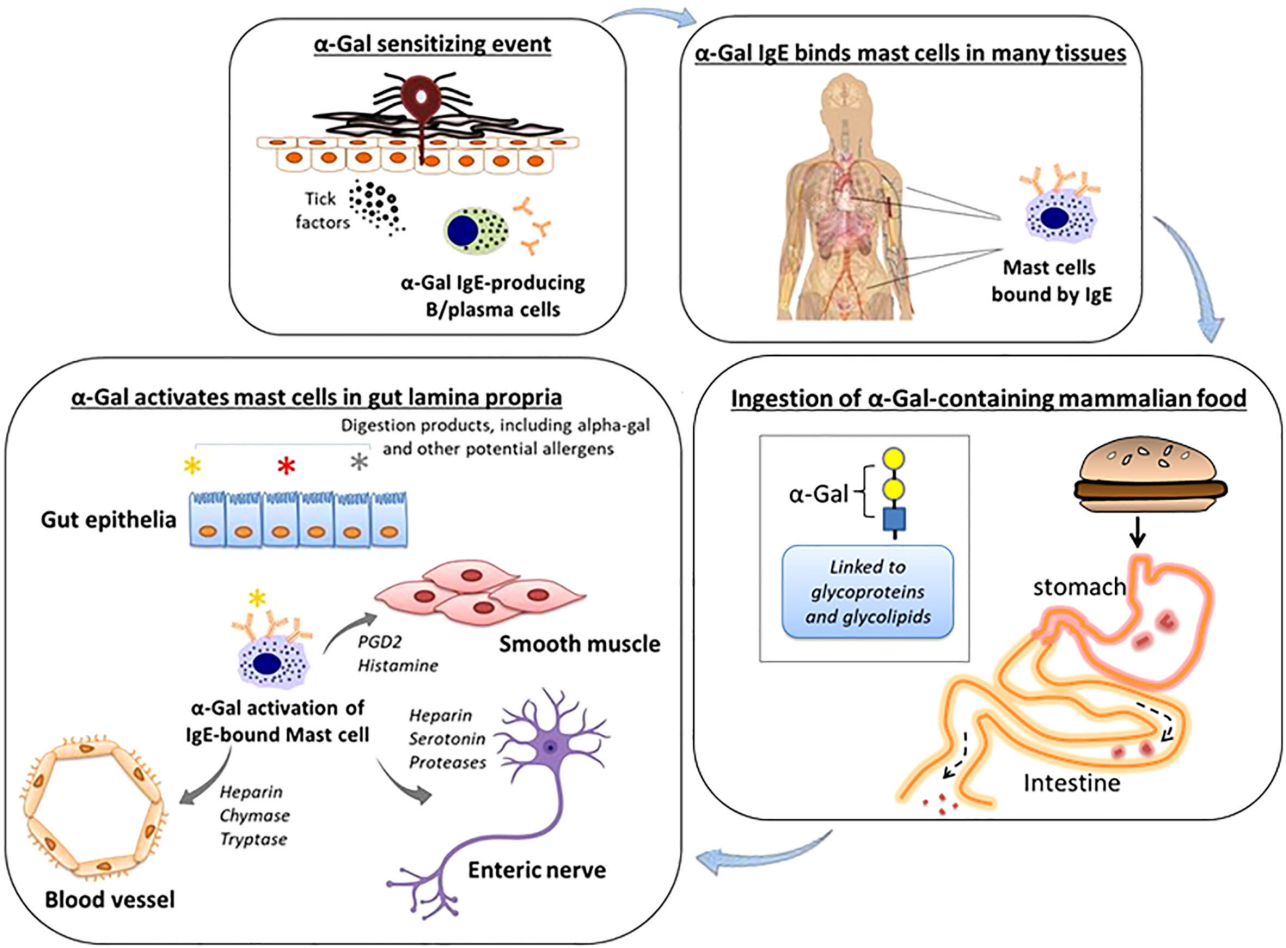
Working model of GI-variant AGS. Tick bites, particularly from *A. americanum*, can induce IgE specific for α-Gal. These IgE antibodies in turn can arm mast cells, including mast cells residing in the gastrointestinal lamina propria, by binding *via* high affinity FcϵR1. Subsequent oral consumption of α Gal-containing mammalian food leads to IgE-dependent activation of mast cells. Cross-talk between mast cells and adjacent blood vessels, smooth muscle and enteric nerves contributes to gastrointestinal symptoms.

IgE to a specific food allergen as a cause of isolated GI symptoms is consistent with a recent study in mice in which specific IgE and mast cells localized to the gut were the cause of meal-induced diarrhea and visceral hypersensitivity (11). Further research is needed, but a working model for GI-AGS involves IgE-armed gut mast cells being activated by α-Gal that reaches the gut lamina propria following the ingestion of mammalian meat, dairy or other products (**Figure 2**). Pro-inflammatory mediators derived from mast cells could drive inflammation, vascular edema, and peristalsis by cross-talk with adjacent cells and tissues, including smooth muscle and enteric nerves (12, 13). The current and previously reported data suggest that low-level α-Gal IgE can be sufficient to cause isolated GI symptoms (9, 10). We would also highlight that the α-Gal IgE levels in GI-AGS in the current study (GM 0.7 kU/L) were similar to the levels in patients with isolated GI symptoms reported by Richards and Richards (Ref 9, median 0.9 kU/L) and Croglio et al. (Ref 10, median 0.6 kU/L).

There are several limitations to consider. This was a retrospective review with no control of dietary α-Gal exclusion, no oral challenges and no numerical quantification of GI symptom scores. Also, differences in timing of onset of GI symptoms that was observed between patients in the allergy group and GI group may be related to variability in history taking and documentation between the groups, or reflective of the lower IgE levels to α-Gal in the patients from the GI clinic. Nevertheless, these results are reflective of the frequency of GI-AGS patients seen in our clinics and experienced by patients in our region.

In conclusion, we have identified and described 75 cases consistent with “GI-variant AGS”. All of these patients presented without classic allergy symptoms such as urticaria, pruritus or anaphylaxis, but were found to be sensitized to α-Gal and reported improvement of their gastrointestinal symptoms on a mammal-restricted diet. GI-AGS symptoms of abdominal pain, diarrhea, and nausea can be confused with many other GI conditions and can be superimposed upon any prior chronic or concomitant condition. Avoidance of mammalian meat is central to management, but many patients require additional elimination of dairy for symptom improvement. Some patients may require exclusion of other sources of α-Gal, such as gelatins, lard, and medications from mammalian sources such as pancreatic replacement enzymes (3, 14, 15). Although none of the patients described here experienced anaphylaxis, providers should be aware that patients with isolated GI symptoms can subsequently experience episodes involving severe systemic reactions. Future studies with placebo-controlled meat challenges will be important to confirm the causal link with α-Gal and provide additional insights into the role of IgE and mast cells in contributing to gastrointestinal morbidity.

## Data availability statement

The raw data supporting the conclusions of this article will be made available by the authors, without undue reservation.

## Ethics statement

The studies involving human participants were reviewed and approved by The University of Virginia Health Sciences IRB approved the study carried out at UVA. The study at Gastroenterology Associates of Central Virginia was exempt because records were extracted by a sole provider and then data was de-identified. Written informed consent from the participants’ legal guardian/next of kin was not required to participate in this study in accordance with the national legislation and the institutional requirements.

## Author contributions

NER, TM, RR and JW carried out chart review. All authors contributed to data analysis and interpretation. NER, TM, RR and JW contributed to the first draft of the manuscript. All authors provided critical feedback on the paper, had full access to the data and accept responsibility for submission.

## Funding

TP-M is a recipient of NIH R37-AI20565. JW is supported by a Faculty Development Award from the American Academy of Allergy Asthma and Immunology.

## Conflict of interest

TP-M and JW received assay support from Thermo-Fisher/Phadia, but not for work related to this project.

The remaining authors declare that the research was conducted in the absence of any commercial or financial relationships that could be construed as a potential conflict of interest.

## Publisher’s note

All claims expressed in this article are solely those of the authors and do not necessarily represent those of their affiliated organizations, or those of the publisher, the editors and the reviewers. Any product that may be evaluated in this article, or claim that may be made by its manufacturer, is not guaranteed or endorsed by the publisher.
